# Analysis of Serum Exosome Metabolites Identifies Potential Biomarkers for Human Hepatocellular Carcinoma

**DOI:** 10.3390/metabo14080462

**Published:** 2024-08-20

**Authors:** Tingting Zhao, Yan Liang, Xiaolan Zhen, Hong Wang, Li Song, Didi Xing, Hui Li

**Affiliations:** 1 College of Pharmacy, Hebei Medical University, Shijiazhuang 050000, China; 2 Hebei Institute of Drug and Medical Device Inspection, Shijiazhuang 050000, China; 3 McCombs Institute for the Early Detection and Treatment of Cancer, Houston, TX 77030, USA; 4 College of Chemistry and Materials Science, Hebei University, Baoding 071002, China

**Keywords:** hepatocellular carcinoma, serum exosomes, non-targeted metabolomics, biomarkers

## Abstract

Currently, the clinical cure rate for primary liver cancer remains low. Effective screening and early diagnosis of hepatocellular carcinoma (HCC) remain clinical challenges. Exosomes are intimately associated with tumor development and their contents have the potential to serve as highly sensitive tumor-specific markers. A comprehensive untargeted metabolomics study was conducted using exosome samples extracted from the serum of 48 subjects (36 HCC patients and 12 healthy controls) via a commercial kit. An ultra-performance liquid chromatography-mass spectrometry (UPLC-MS) strategy was used to identify the metabolic compounds. A total of 18 differential metabolites were identified using the non-targeted metabolomics approach of UPLC-QTOF-MS/MS. Pathway analysis revealed significant alterations in the arachidonic acid metabolism, linoleic acid metabolism, and unsaturated fatty acid metabolism pathways. ROC analysis indicated that three metabolites with AUC values exceeding 0.900 were selected as potential biomarkers: caprylic acid and linoleic acid were upregulated in the HCC group, whereas pentadecanoic acid was downregulated. Linoleic acid, caprylic acid, and pentadecanoic acid are potential biomarkers for diagnosing HCC. The significant alterations in these three metabolic pathways offer new insights into the mechanisms underlying HCC formation and progression.

## 1. Introduction

The incidence of primary liver cancer is rising globally, with hepatocellular carcinoma accounting for over 90% of cases, and China has the highest incidence and mortality rates of liver cancer worldwide [1]. Early-stage HCC often presents without specific clinical symptoms or signs, or only with those resembling cirrhosis, and by the time obvious clinical symptoms and signs appear, the cancer is usually in the middle or late stages, significantly impacting the cure rate and prognosis [2].

Currently, the primary bases for confirming the diagnosis of HCC include serologic markers (alpha-fetoprotein (AFP)), imaging examinations (ultrasound, CT, MRI), and pathological tissue biopsies. However, when the diameter of the cancer lesion is less than 3.0 cm, the sensitivity of AFP is only 25–50% [3,4]. On the one hand, there is a risk of missed diagnosis due to variations in doctors’ expertise and, on the other hand, imaging examinations require long-term, periodic follow-ups, which is time-consuming, costly, and leads to low compliance [5]. A pathologic tissue biopsy, as a confirmatory test, is not suitable for disease screening due to its invasiveness. Therefore, there is an urgent need to develop new, specific early diagnostic methods.

In this study, exosomes are characterized as nanosized vesicles, spanning diameters between 30 and 150 nm, which encapsulate a diverse cargo of DNA, RNA, proteins, and other bioactive small molecules [6]. These vesicles act as pivotal mediators of intercellular communication, facilitating the transfer of vital information [7]. Notably, the composition of exosomal contents undergoes dynamic alterations during the onset and progression of tumorigenesis. Specifically, exosomal LysoPC (22:0), PC (P-14:0/22:2), and PE (16:0/18:1) have been implicated in correlating with CA19-9 levels, tumor staging, and size in pancreatic cancer [8], while PG (34:1)-H lipids exhibit significant differences in ovarian cancer contexts [9]. Furthermore, the membrane lipidome of exosomes derived from colon cancer patients is enriched in polyunsaturated fatty acids, particularly omega-6 fatty acids, suggesting a distinct metabolic signature [10]. Consequently, the differential lipid profiles within exosomes hold promise as diagnostic biomarkers for cancer, underscoring the need for comprehensive serum exosome-based metabolic profiling in HCC patients. This approach offers a novel lens for monitoring HCC progression and sheds light on the exploration of exosome-mediated metabolic pathways pertinent to HCC.

Metabolomics, a field that concurrently examines metabolic processes qualitatively and quantitatively over a defined physiological period, is instrumental in unraveling the intricacies of organismal or cellular metabolism [11]. Metabolites, being the end products of cellular processes, are believed to harbor extensive information that can predictively map phenotypic traits [12]. Metabolomics offers several advantages in screening for tumor markers, including high sensitivity, high throughput, non-invasiveness, and low cost. Ultra-high-performance liquid chromatography-tandem mass spectrometry (UPLC-MS/MS) is widely utilized for its high accuracy and sensitivity in histological analyses [13,14].

Based on UPLC-ESI-MS/MS analysis and multivariate statistical analysis, the primary objective of this study was to identify highly sensitive biomarkers for hepatocellular carcinoma. A total of 48 exosome samples derived from serum were analyzed, comprising healthy controls and HCC patient groups.

## 2. Materials and Methods

### 2.1. Collection of Clinical Samples

A total of 36 patients diagnosed with HCC and 12 healthy volunteers were meticulously recruited from the Bethune International Peace Hospital. A comprehensive set of 48 serum samples was systematically obtained, with each being promptly centrifuged within four hours of collection to ensure sample integrity. These samples were then carefully stored in an ultra-low-temperature freezer at −80 °C, preserving them for subsequent analysis. This study was approved by the Ethics Committee of the Bethune International Peace Hospital (Table 1).

### 2.2. Exosome Isolation and Characterization

After centrifuging 100 μL of serum at 3000× *g* for 15 min at 4 °C, the supernatant was collected and 25.2 μL of ExoQuick-TC^TM^ reagent was added. For exosome precipitation, the mixture was centrifuged at 1500× *g* for 30 min at 4 °C to separate the supernatant, followed by a further 5-min centrifugation at 1500× *g* to remove any residual liquid. The sample was then prepared for injection to ensure optimal analysis. From each serum sample, 10 μL was pooled to create quality control (QC) samples, which were processed in parallel and identically to the study samples.

### 2.3. Extraction of Exosome Metabolites

To the exosome sample, 0.5 mL of acetonitrile was added and the mixture was vortexed for 30 s with vigorous shaking. The mixture was then sonicated for 45 min and incubated at −20 °C overnight to precipitate proteins in the sample. The samples were centrifuged at 12,000× *g* for 10 min and the supernatant was carefully removed, dried under a stream of nitrogen, and reconstituted with 50 μL of acetonitrile. All samples were stored at −80 °C until analysis.

### 2.4. UPLC-Q-TOF-MS/MS Analysis

Chromatographic separation was conducted using ultra-performance liquid chromatography under optimized conditions. The Waters HSS T3 column (150 × 3 mm, 1.8 μm) was employed and maintained at 40 °C, with mobile phases comprising (A) 0.1% formic acid in water and (B) acetonitrile in the positive mode. For negative ionization mode, mobile phases were adjusted to (A) 6.5 mM ammonia bicarbonate in water and (B) a 5% water–methanol mixture. The elution gradient was tailored as follows: 0–4 min, 2–40% B; 4–12 min, 40–100% B; 12–16 min, 100% B; and 16–20 min, 2% B, all at a constant flow rate of 0.30 mL/min. The injection volume was standardized at 3 μL, with the autosampler tray temperature maintained at 4 °C.

Serum exosome samples were analyzed using a Triple TOF 6600+ mass spectrometer (Sciex, Framingham, MA, USA) in ESI (+/−) mode, operating in IDA mode. The scanning range was set to encompass *m*/*z* 50–1200 Da, with positive/negative ion source voltages adjusted to 5500 V/−4500 V, respectively. The collision energy was set at 10 V; de-clustering voltage at 80 V; and the gas parameters were optimized with a curtain gas of 30 psi, sheath gas of 50 psi, and heating gas of 50 psi. The ion source temperature was uniformly maintained at 500 °C. The CE ± CES used to acquire the secondary mass spectra was 35 ± 15 V.

### 2.5. Data Analysis

Data preprocessing was performed using MS-DIAL ver.4.9. for peak extraction, the automatic checking of peak alignment, normalization, and the background deduction of mass spectrometry raw data. The MS/MS fragmentation patterns and retention times of the target endogenous metabolites were searched in the METLIN database, HMDB database, and KEGG database, and the results of metabolite identification were obtained by comparing the MS/MS fragmentation patterns and retention times of the target endogenous metabolites with those of the standards.

Statistical analyses included unpaired *t*-tests, fold-change assessments, unsupervised principal component analysis (PCA), and supervised orthogonal partial least squares discriminant analysis (OPLS-DA). The outcomes of the multivariate analyses were visualized using score plots, revealing the overall distribution trends of the samples. OPLS-DA, an extension of the traditional PLS-DA method, was employed for the classification and discrimination of two or more data classes. Following the identification of differentially expressed metabolites using OPLS-DA, the model’s reliability was evaluated through cross-validation and permutation testing.

Significantly different metabolites were selected based on screening criteria of *p* < 0.05, fold change (FC) values > 2 or <0.5, and variable importance in projection (VIP) scores > 1. Receiver operating characteristic (ROC) curves were plotted for each subject and the area under each curve was calculated to assess the predictive performance of the differential metabolites.

Finally, the differential metabolites were subjected to metabolic pathway analysis using the MetaboAnalyst online platform.

## 3. Results

### 3.1. Reliability of the Analytical Method

**Figure 1:**
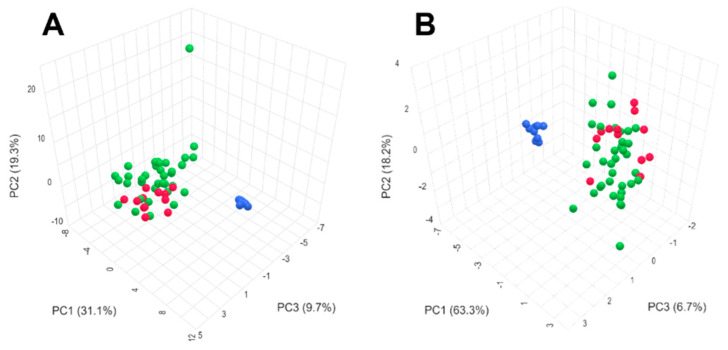
PCA score plot (**A**) Positive pattern of exosome samples (**B**) Negative pattern of exosome samples, with the HCC group in green, the HC group in red, and the QC group in blue.

System stability was maintained by injecting one QC sample after every five experimental samples throughout the entire sample batch. PCA was used to evaluate the system’s stability and reproducibility. Representative PCA score plots for all experimental samples and QC samples are shown in Figure 1. The QC samples clustered tightly in the score plots, indicating that the system exhibited high stability and reproducibility over the course of the experiment.

### 3.2. Differential Metabolite Screening

**Figure 2:**
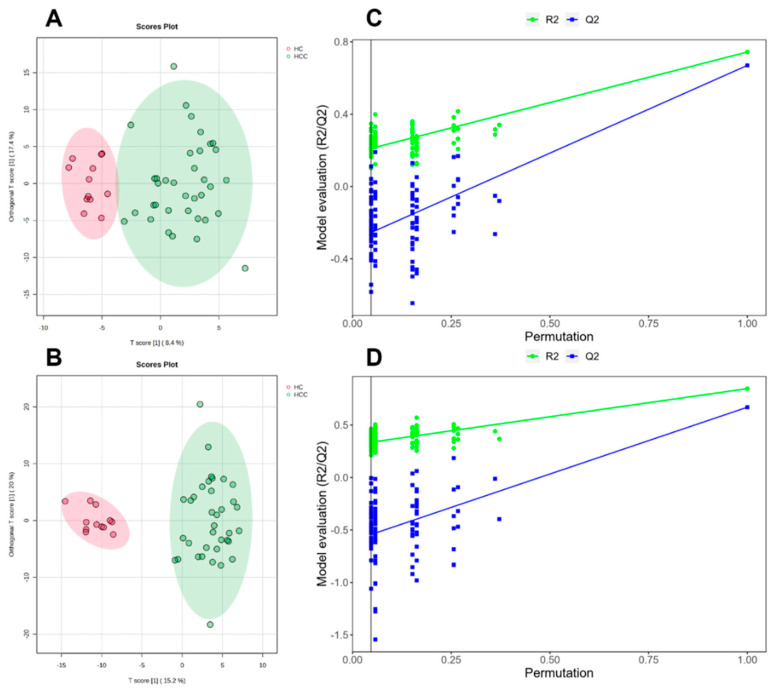
OPLS−DA score plot (**A**) Positive pattern of exosome samples (**B**) Negative pattern of exosome samples. Permutation (**C**) Positive pattern of exosome samples (**D**) Negative pattern of exosome samples.

The supervised method OPLS-DA was used for further modeling analysis. The classification effect of the two groups was more significant, as seen by the OPLS-DA plot (Figure 2). R^2^Y = 0.90 and Q^2^Y = 0.67, with R^2^Y close to 1, indicating a better explanation rate. The results of the model replacement test are shown in Figure 2; in both models, the left-side R^2^ intercept and Q^2^ intercept are lower than the right-side values and the Q^2^ intercept is lower than 0. According to the above parameters, it indicates that there is no overfitting phenomenon in the model and, therefore, the metabolic model is stable and reliable.

The VIP values of the first two principal components of the OPLS-DA model were analyzed by multivariate analysis and combined with *p*-values and fold changes to screen for differential metabolites. The screening criteria were as follows: (1) *p*-value < 0.05; (2) VIP value > 1.00; (3) FC value > 2 or <0.5. Using UPLC-Q-TOF-MS/MS, a total of 18 significant differential metabolites were identified between the two groups, which are detailed in Table 2. Relatively higher concentrations of taurochenodeoxycholic acid, tauroursodeoxycholic acid, taurodeoxycholic acid, linoleic acid, and caprylic acid were observed in the HCC group compared to the HC group. Conversely, the healthy group showed relatively higher concentrations of oleic acid, two phosphatidylcholines, four epoxyeicosatrienoic acids (EETs), and five hydroxyeicosatetraenoic acids (HETEs).

**Figure 3:**
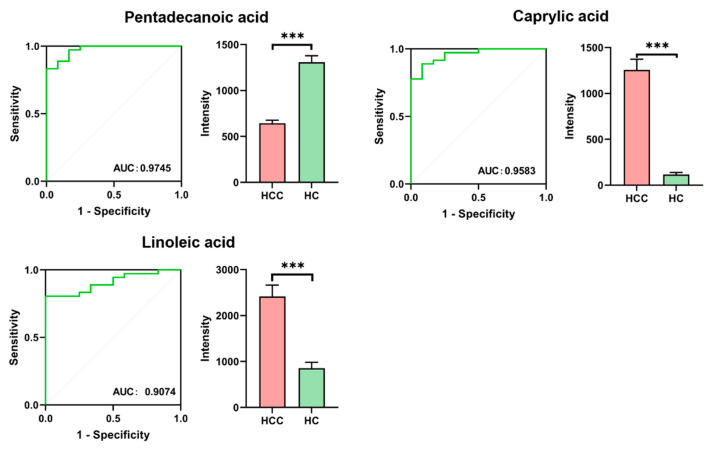
ROC analysis results of three potential biomarkers and the levels of potential markers in the HCC group and HC group, *** *p* < 0.05.

The accuracy of the metabolites in distinguishing hepatocellular carcinoma patients from healthy individuals was determined by the area under the ROC curve (AUC) values. Based on the results of ROC curves, metabolites with an AUC > 0.90 were selected as potential biomarkers. A total of three metabolites were identified: linoleic acid, caprylic acid, and pentadecanoic acid. Box plots were generated to visualize the relative levels of these potential markers between the two groups (Figure 3).

### 3.3. Metabolic Pathway Analysis

**Figure 4:**
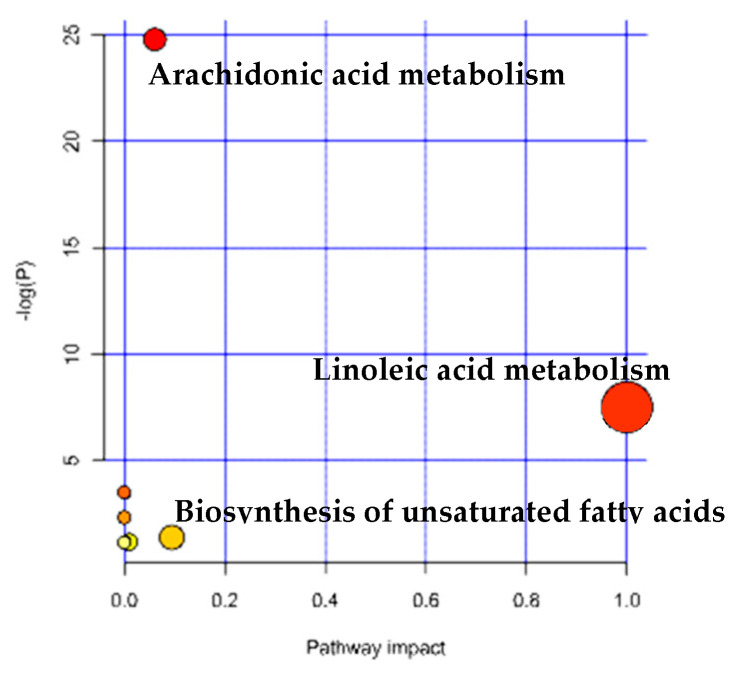
Bubble chart for differential metabolite pathway analysis in HCC.

Eighteen differential metabolites were subjected to metabolite pathway analysis using MetaboAnalyst 5.0 to analyze the alteration of metabolic pathways in humans with hepatocellular carcinoma (Figure 4) Pathway topology analysis provided the *x*−axis with pathway impact values, while pathway analysis provided the *y*−axis with -log(*p*) values. Pathways with a *p*−value < 0.05 were considered to have a high impact (Table 3).

## 4. Discussion

The MetaboAnalyst 5.0 pathway analysis and literature review revealed significant changes (*p* < 0.05) in several key metabolic pathways, including arachidonic acid metabolism, linoleic acid metabolism, and unsaturated fatty acid biosynthesis.

### 4.1. Arachidonic Acid Metabolism

Pathway analysis of differential metabolites revealed that, compared to the HC group, the HCC group showed the most significant changes in the metabolism of arachidonic acid (AA), linoleic acid (LA), and unsaturated fatty acids (*p* < 0.001). We observed that the levels of nine metabolites involved in arachidonic acid metabolism underwent significant decreases, with generally similar magnitudes. AA can be metabolized into two types of metabolites by different members of the CYP2A family [15]. Additionally, ω-Hydroxylase enzymes convert AA to hydroxyeicosatetraenoic acids (HETEs) while cyclooxygenases convert AA to epoxyeicosatrienoic acids (EETs) [16]. Overall, HCC reduces the enzymatic pathway metabolism of arachidonic acid, which may be related to altered CYP family expression. A typical role for CYP2A6 is to decrease the production of EETs and increase the production of 20-HETE in AA metabolism. Additionally, 20-HETE promotes M1 macrophage polarization, stimulates pro-inflammatory cytokine production, and enhances macrophage phagocytosis, thereby contributing to tumor killing. When CYP2A6 is reduced in HCC, macrophage polarization is affected, creating a tumor microenvironment conducive to tumor growth. Abnormal expression of CYP2A6 may disrupt the balance between 20-HETE and EETs, leading to a reduced conversion of AA to 20-HETE, which is consistent with the decrease in 20-HETE observed in this study. Our study also found a similar decrease in EETs, which may be due to decreased metabolism by other enzyme pathways [15]. A study demonstrated that during the progression of HCC, the expression of CYP2C8 and CYP2C9 significantly decreased (Kruskal–Wallis, *p* < 0.001). A significant decrease in CYP2C9 expression led to a decrease in EET levels [17], which aligns with our findings.

CYP-produced EETs modulate inflammation, angiogenesis, and vascular tone. In mice with non-alcoholic steatohepatitis, EETs have a protective effect [18]. It has been demonstrated that EETs, acting as paracrine molecules within the microvascular endothelium, play a critical role in liver regeneration [19,20]. EETs’ pro-angiogenic effects could explain their association with tumor growth. Several studies have shown that increasing 14,15-EET promotes tumor growth and metastasis via cell invasion when soluble epoxide hydrolase (sEH) is inhibited [21]. By administering 15-HETE exogenously, it has been demonstrated that blocking the 15-LOX-1 pathway with selective inhibitors or siRNA causes growth arrest and apoptosis in HCC cells [22]. It is evident that the metabolites EET and HETE are closely associated with the presence of HCC. We speculate whether it is possible to interfere with the metabolic pathways of EETs and HETEs to protect and repair normal hepatocytes and inhibit the development of hepatocellular carcinoma. Preemptive monitoring of the metabolic levels of EETs and HETEs may be helpful in achieving the early diagnosis of hepatocellular carcinoma.

### 4.2. Linoleic Acid and Unsaturated Fatty Acid Metabolism

Linoleic acid and oleic acid were involved in both pathways and displayed statistically different metabolic levels (*p* < 0.05, FC value > 2 or <0.5, and VIP > 1). In the present study, linoleic acid had an AUC value > 0.9 and its metabolic level was upregulated compared to the HC group. It has been found that linoleic acid interferes with the immune response in the liver, promoting hepatocellular carcinoma development [23]. This finding is consistent with the results of the current study. Linoleic acid upregulates carnitine palmitoyl transferase 1 (CPT1) to induce apoptosis in CD4+ T cells, disrupting mitochondrial function, while also increasing fatty acid desaturase 2 (FADS2) expression in tumors [23,24,25]. Furthermore, linoleic acid interferes with the immune response in mice with non-alcoholic steatohepatitis (NASH). Normal livers have the potential to progress to chronic liver disease (from hepatic steatosis to NASH) and, ultimately, to primary liver cancer [26]. We hypothesize that altered linoleic acid metabolism may be one of the pathways leading to HCC. Whether linoleic acid metabolism levels are statistically different in HCC and NASH remains uncertain and should be examined in a separate cohort.

Oleic acid (OA), as a monounsaturated fatty acid, is one of the most abundant fatty acids. Studies have shown that the metastatic potential of cancer cells is associated with genes involved in fatty acid synthesis and intracellular lipid storage [27,28]. OA regulates cell death by altering lipid metabolism or changing membrane lipid composition [29,30]. Specifically, OA induces lipid accumulation in hepatocyte cell lines and high levels of OA reduce viability, proliferation, migration, and invasive behaviors in HCC cell lines, potentially leading to cell death [31]. Consistent with previous studies, our metabolomics analysis showed that the HCC group had metabolically downregulated OA compared to the HC group. Based on these findings, targeting OA metabolism pathways could potentially be used as a therapeutic strategy for HCC and other forms of cancer.

## 5. Conclusions

Our study comprehensively analyzed metabolic alterations in serum exosomes from patients with primary hepatocellular carcinoma using UPLC-MS-based metabolomics and metabolic pathway analysis. Among the 18 crucial differential metabolites identified, linoleic acid, octanoic acid, and pentadecanoic acid exhibited high area under the curve values. In pathway analysis matching, these key differential metabolites, reductions in arachidonic acid derivatives, elevations in linoleic acid metabolic levels, and downregulations in OA metabolic levels were observed in the HCC group, offering novel insights into the mechanisms underlying HCC formation and progression.

Notably, the relatively small number of clinical samples in this study may have impacted data integrity. Metabolite identification relied on three databases, yet, due to the incompleteness of these databases, a substantial proportion of ion feature peaks remained unmatched, resulting in a compound identification rate of approximately 15%. This limitation represents a current bottleneck in metabolomics research. Additionally, the incomplete coverage of metabolic pathways by the KEGG database hindered the assignment of many identified differential metabolites to specific pathways, necessitating an extensive literature review to explore the mechanisms of action of these crucial metabolites in HCC development, a time-consuming endeavor.

Regarding potential biomarkers associated with HCC development, functional validation can be conducted at the cellular or animal level, involving the addition or subtraction of small molecule metabolites or the knock-out or knock-in of key proteins and enzymes that influence these metabolites, to evaluate their impact on the growth and metastasis of HCC cells or tumors in animals. Addressing these limitations constitutes a crucial direction for our future work.

## Figures and Tables

**Table 1:** Clinical characteristics of the subjects.

Characteristics	HC Group	HCC Group
Number	12	36
Gender (male/female)	9/3	30/6
Age (years)	49.2 ± 7.5	59.2 ± 8.2
AFP (ng/mL)	3.0 ± 1.5	106.8 ± 126.3
CEA (ng/mL)	1.8 ± 0.5	3.5 ± 1.8

HC, health control.

**Table 2:** Differential metabolites in the exosomes and serum of HCC patients.

Metabolite	VIP	FC	*p*	Pathway
Linoleic acid ↑	1.39	2.02	<0.001	Biosynthesis of unsaturated fatty acids Linoleic acid metabolism
Caprylic acid ↑	1.50	10.55	<0.001	Fatty acid biosynthesis
Taurochenodeoxycholic acid ↑	1.29	17.92	0.030	Primary bile acid biosynthesis
Tauroursodeoxycholic acid ↑	1.34	17.92	0.034	NA
Taurodeoxycholic acid ↑	1.36	17.92	0.028	NA
Pentadecanoic acid ↓	1.13	0.49	<0.001	NA
Hexacosanoic acid ↓	3.78	0.22	0.009	NA
PC(16:0/20:4) ↓	2.57	0.40	0.002	NA
PC(18:2/18:2) ↓	2.64	0.40	0.003	NA
Oleic acid ↓	1.31	0.47	0.023	Biosynthesis of unsaturated fatty acids
5,6-EET ↓	1.84	0.29	0.002	Arachidonic acid metabolism
14,15-EET ↓	1.85	0.29	0.002	NA
16(R)-HETE ↓	1.81	0.29	0.002	NA
8,9-EET ↓	1.87	0.29	0.002	Arachidonic acid metabolism
11,12-EET ↓	1.86	0.29	0.002	Arachidonic acid metabolism
20-HETE ↓	1.85	0.29	0.002	Arachidonic acid metabolism
15(S)-HETE ↓	1.85	0.29	0.002	Arachidonic acid metabolism
19(S)-HETE ↓	1.84	0.29	0.002	Arachidonic acid metabolism

The ↑ indicates that the metabolite is upregulated in the HCC group, and the ↓ indicates that it is downregulated.

**Table 3:** Metabolic pathway analysis results.

Pathway	Total	Hits	Raw P	Impact
Arachidonic acid metabolism	36	8	<0.001	0.06
Linoleic acid metabolism	5	2	<0.001	1.00
Biosynthesis of unsaturated fatty acids	36	2	0.030	0.01
Primary bile acid biosynthesis	46	1	0.304	0.01
Fatty acid biosynthesis	47	1	0.310	<0.01

## Data Availability

The data that support the findings of this study are available from the corresponding author, upon reasonable request.

## References

[1] McGlynn K.A., Petrick J.L., London W.T. (2015). Global epidemiology of hepatocellular carcinoma: An emphasis on demographic and regional variability. Clin. Liver Dis..

[2] Sakamoto M. (2009). Early HCC: Diagnosis and molecular markers. J. Gastroenterol..

[3] Wolf E., Rich N.E., Marrero J.A., Parikh N.D., Singal A.G. (2021). Use of Hepatocellular Carcinoma Surveillance in Patients with Cirrhosis: A Systematic Review and Meta-Analysis. Hepatology.

[4] Tzartzeva K., Obi J., Rich N.E., Parikh N.D., Marrero J.A., Yopp A., Waljee A.K., Singal A.G. (2018). Surveillance Imaging and Alpha Fetoprotein for Early Detection of Hepatocellular Carcinoma in Patients with Cirrhosis: A Meta-analysis. Gastroenterology.

[5] Marrero J.A. (2020). Surveillance for Hepatocellular Carcinoma. Clin. Liver Dis..

[6] Doyle L.M., Wang M.Z. (2019). Overview of Extracellular Vesicles, Their Origin, Composition, Purpose, and Methods for Exosome Isolation and Analysis. Cells.

[7] Kumar A., Deep G. (2020). Exosomes in hypoxia-induced remodeling of the tumor microenvironment. Cancer Lett..

[8] Tao L., Zhou J., Yuan C., Zhang L., Li D., Si D., Xiu D., Zhong L. (2019). Metabolomics identifies serum and exosomes metabolite markers of pancreatic cancer. Metabolomics.

[9] Cheng L., Zhang K., Qing Y., Li D., Cui M., Jin P., Xu T. (2020). Proteomic and lipidomic analysis of exosomes derived from ovarian cancer cells and ovarian surface epithelial cells. J. Ovarian Res..

[10] Scavo M.P., Depalo N., Tutino V., De Nunzio V., Ingrosso C., Rizzi F., Notarnicola M., Curri M.L., Giannelli G. (2020). Exosomes for Diagnosis and Therapy in Gastrointestinal Cancers. Int. J. Mol. Sci..

[11] Fiehn O. (2002). Metabolomics—The link between genotypes and phenotypes. Plant Mol. Biol..

[12] Schrimpe-Rutledge A.C., Codreanu S.G., Sherrod S.D., McLean J.A. (2016). Untargeted Metabolomics Strategies—Challenges and Emerging Directions. J. Am. Soc. Mass. Spectrom..

[13] Nassar A.F., Wu T., Nassar S.F., Wisnewski A.V. (2017). UPLC-MS for metabolomics: A giant step forward in support of pharmaceutical research. Drug Discov. Today.

[14] Zhang J., Tang C., Oberly P.J., Minnigh M.B., Achilles S.L., Poloyac S.M. (2019). A sensitive and robust UPLC-MS/MS method for quantitation of estrogens and progestogens in human serum. Contraception.

[15] Jiang T., Zhu A.S., Yang C.Q., Xu C., Yang D., Lou Z., Zhang G. (2021). Cytochrome P450 2A6 is associated with macrophage polarization and is a potential biomarker for hepatocellular carcinoma. FEBS Open Bio.

[16] Zeldin D.C. (2001). Epoxygenase pathways of arachidonic acid metabolism. J. Biol. Chem..

[17] Wang Q., Tang Q., Zhao L., Zhang Q., Wu Y., Hu H., Liu L., Liu X., Zhu Y., Guo A. (2020). Time serial transcriptome reveals Cyp2c29 as a key gene in hepatocellular carcinoma development. Cancer Biol. Med..

[18] Wang X., Li L., Wang H., Xiao F., Ning Q. (2019). Epoxyeicosatrienoic acids alleviate methionine-choline-deficient diet-induced non-alcoholic steatohepatitis in mice. Scand. J. Immunol..

[19] Panigrahy D., Kalish B.T., Huang S., Bielenberg D.R., Le H.D., Yang J., Edin M.L., Lee C.R., Benny O., Mudge D.K. (2013). Epoxyeicosanoids promote organ and tissue regeneration. Proc. Natl. Acad. Sci. USA.

[20] Zhang G., Kodani S., Hammock B.D. (2014). Stabilized epoxygenated fatty acids regulate inflammation, pain, angiogenesis and cancer. Prog. Lipid Res..

[21] Panigrahy D., Edin M.L., Lee C.R., Huang S., Bielenberg D.R., Butterfield C.E., Barnés C.M., Mammoto A., Mammoto T., Luria A. (2012). Epoxyeicosanoids stimulate multiorgan metastasis and tumor dormancy escape in mice. J. Clin. Investig..

[22] Ma J., Zhang L., Zhang J., Liu M., Wei L., Shen T., Ma C., Wang Y., Chen Y., Zhu D. (2013). 15-lipoxygenase-1/15-hydroxyeicosatetraenoic acid promotes hepatocellular cancer cells growth through protein kinase B and heat shock protein 90 complex activation. Int. J. Biochem. Cell Biol..

[23] Ma C., Kesarwala A.H., Eggert T., Medina-Echeverz J., Kleiner D.E., Jin P., Stroncek D.F., Terabe M., Kapoor V., ElGindi M. (2016). NAFLD causes selective CD4(+) T lymphocyte loss and promotes hepatocarcinogenesis. Nature.

[24] Muir K., Hazim A., He Y., Peyressatre M., Kim D.-Y., Song X., Beretta L. (2013). Proteomic and lipidomic signatures of lipid metabolism in NASH-associated hepatocellular carcinoma. Cancer Res..

[25] Brown Z.J., Fu Q., Ma C., Kruhlak M., Zhang H., Luo J., Heinrich B., Yu S.J., Zhang Q., Wilson A. (2018). Carnitine palmitoyltransferase gene upregulation by linoleic acid induces CD4+ T cell apoptosis promoting HCC development. Cell Death Dis..

[26] Pfister D., Núñez N.G., Pinyol R., Govaere O., Pinter M., Szydlowska M., Gupta R., Qiu M., Deczkowska A., Weiner A. (2021). NASH limits anti-tumour surveillance in immunotherapy-treated HCC. Nature.

[27] Sánchez-Martínez R., Cruz-Gil S., Gómez de Cedrón M., Alvarez-Fernandez M., Vargas T., Molina S., García B., Herranz J., Moreno-Rubio J., Reglero G. (2015). A link between lipid metabolism and epithelial-mesenchymal transition provides a target for colon cancer therapy. Oncotarget.

[28] Giampietri C., Tomaipitinca L., Scatozza F., Facchiano A. (2020). Expression of Genes Related to Lipid Handling and the Obesity Paradox in Melanoma: Database Analysis. JMIR Cancer.

[29] Fontana A., Spolaore B., Polverino de Laureto P. (2013). The biological activities of protein/oleic acid complexes reside in the fatty acid. Biochim. Biophys. Acta.

[30] Ning J., Zhao C., Chen J.X., Liao D.F. (2019). Oleate inhibits hepatic autophagy through p38 mitogen-activated protein kinase (MAPK). Biochem. Biophys. Res. Commun..

[31] Giulitti F., Petrungaro S., Mandatori S., Tomaipitinca L., de Franchis V., D’Amore A., Filippini A., Gaudio E., Ziparo E., Giampietri C. (2021). Anti-tumor Effect of Oleic Acid in Hepatocellular Carcinoma Cell Lines via Autophagy Reduction. Front. Cell Dev. Biol..

